# Bio-inspired, Moisture-Powered Hybrid Carbon Nanotube Yarn Muscles

**DOI:** 10.1038/srep23016

**Published:** 2016-03-14

**Authors:** Shi Hyeong Kim, Cheong Hoon Kwon, Karam Park, Tae Jin Mun, Xavier Lepró, Ray H. Baughman, Geoffrey M. Spinks, Seon Jeong Kim

**Affiliations:** 1 Center for Self-powered Actuation and Department of Biomedical Engineering, Hanyang University, Seoul 133-791, South Korea; 2 The Alan G. MacDiarmid NanoTech Institute, University of Texas at Dallas, Richardson, TX 75083, USA; 3 Intelligent Polymer Research Institute, ARC Centre of Excellence for Electromaterials Science, University of Wollongong, Wollongong, New South Wales 2522, Australia

## Abstract

Hygromorph artificial muscles are attractive as self-powered actuators driven by moisture from the ambient environment. Previously reported hygromorph muscles have been largely limited to bending or torsional motions or as tensile actuators with low work and energy densities. Herein, we developed a hybrid yarn artificial muscle with a unique coiled and wrinkled structure, which can be actuated by either changing relative humidity or contact with water. The muscle provides a large tensile stroke (up to 78%) and a high maximum gravimetric work capacity during contraction (2.17 kJ kg^−1^), which is over 50 times that of the same weight human muscle and 5.5 times higher than for the same weight spider silk, which is the previous record holder for a moisture driven muscle. We demonstrate an automatic ventilation system that is operated by the tensile actuation of the hybrid muscles caused by dew condensing on the hybrid yarn. This self-powered humidity-controlled ventilation system could be adapted to automatically control the desired relative humidity of an enclosed space.

Artificial muscles that transform input electrical, thermal, or chemical energy to the mechanical energy of tensile contraction, torsional rotation, or bending have attracted enormous interest^1^,^2^,^3^,^4^. Natural phenomena, such as the opening of pine cones^5^ and seed dispersal^6^ in response to changes in humidity, have inspired the development of self-powered hygromorph artificial muscles capable of generating useful mechanical^7^,^8^,^9^,^10^,^11^,^12^,^13^,^14^,^15^ and electrical energy^16^,^17^,^18^,^19^,^20^. Since most of these moisture driven artificial muscles produce bending^8^,^9^,^10^,^11^,^12^,^13^,^14^,^15^,^17^,^18^,^19^ or torsional^15^,^19^ rotation, they are difficult to upscale by configuring multiple actuators to work in parallel. Sahin’s group recently developed a hygromorph tensile actuator^16^ that could be upscaled, and demonstrated the use of this actuator for evaporation-driven engines and mechano-electrical energy generators. However, the realized gravimetric work capacity (0.017 kJ kg^−1^)^16^ was much smaller than demonstrated during contraction for thermally powered hybrid carbon nanotube (CNT) yarn muscles (1.36 kJ kg^−1^)^3^ and twisted polymer fibre muscles (2.48 kJ kg^−1^)^4^.

To develop hygromorph tensile artificial muscle that provide large work capacity, we employed the technique of biscrolling to generate twisted yarn structures that combine both coiled and wrinkled structural features. Previous hybrid yarn tensile artificial muscles have been constructed by biscrolling a guest, or by such methods as infiltrating a paraffin wax guest into a coiled yarn^3^. Changing the volume of the guest by heating the coiled yarn generates large tensile contractions for yarns made purely by yarn twisting, so that the handedness of yarn twist and coiling are identical^3^. Given that actuation of a coiled hybrid yarn muscle depends on volume-change-driven dimensional changes, which produce muscle length contraction, yarn diameter increase, and yarn untwist, we sought to enhance muscle performance by introducing a wrinkled structure that was bio-inspired by *Bacillus* spores. It is well known that the wrinkled coat of the spore can enable a large volume change of 12% by unfolding of the wrinkled structure when water is absorbed^17^,^21^. Here, we developed a bio-inspired hybrid yarn artificial muscle (HYAM) with a coiled and wrinkled structure by highly twisting a CNT sheet stack that incorporated a hydrophilic poly(diallyldimethylammonium chloride) (PDDA) guest. Changes to the morphology of the PDDA/CNT HYAM can be driven by absorbing water or by a change in the ambient relative humidity (RH). The water-driven HYAM provides a large tensile stroke (up to 78%), a large gravimetric work capacity (2.17 kJ kg^−1^) and high volumetric work capacities (1.8 MJ m^−3^).

## Results

### Preparation of PDDA/CNT hybrid yarn artificial muscle with coiled and wrinkled structure

**Figure 1:**
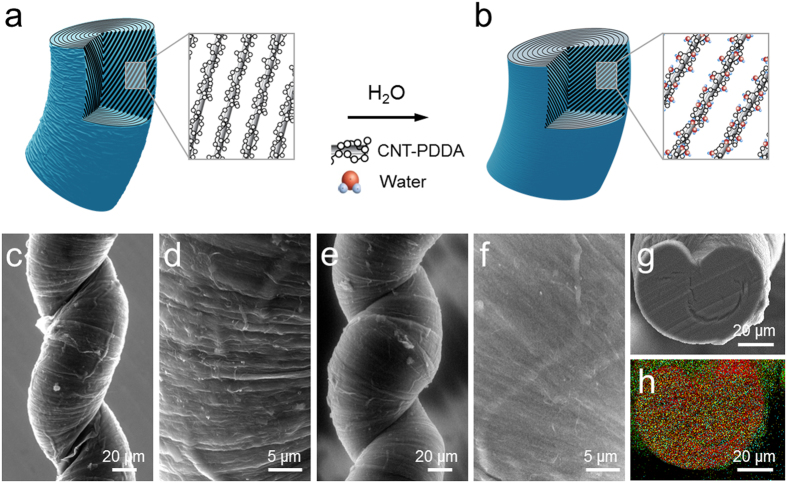
Structure of the HYAM. Schematic diagram showing the change in structure of part of the HYAM (**a**) before and (**b**) after water absorption. (**c**) SEM image of the HYAM at 10% RH. (**d**) Higher magnification SEM image of (**c**) showing the winkled structure on the surface of the HYAM. (**e**) SEM image of the HYAM at 90% RH. The yarn diameter of the HYAM of (**e**) is 30% larger than the yarn diameter of the HYAM of (**d**). (**f**) Higher magnification of the SEM image of (**e**) showing unfolded surface. (**g**) Cross-sectional SEM image of the HYAM prepared by FIB cutting. (**h**) Elemental mapping by EDAX over the image (see Figure S1; red, yellow, and green spots correspond to C, Cl, and O, respectively). The HYAMs used in (**b**–**g**) include 30 wt% PDDA.

The schematic diagrams shown in Fig. 1a,b represent a water-free (Fig. 1a) and a water-absorbed (Fig. 1b) coiled PDDA/CNT HYAM. Given that PDDA is well known as a material that absorbs water from air, and considering that it can easily attached to CNTs by π–π interaction^22^, we selected this material for study. HYAMs were fabricated by simply inserting twist into a carbon nanotube sheet stack (the host) containing fully hydrated infiltrated 30 wt% PDDA guest (see the “Sample preparation” section in Method). Unlike reported for biscrolling in the dry state^3^,^23^, coiling occurs as a process that balances the anisotropic stresses needed for extruding water from the PDDA with the applied anisotropic stresses generated by high twist insertion under isobaric load. Hence, twist insertion, which decreases yarn volume, partially extrudes water from the PDDA during fabrication of a coiled yarn muscle. Despite the high loading of PDDA in the muscle (30 wt%), the dry HYAM had an ultimate strength of 127 MPa, a breaking strain of 150%, and an elastic modulus of 32 MPa (Figure S1).

After coiling, as the water in the HYAM evaporates, the twisting force increases as the volume of the HYAM decreases. The resulting structure for the coiled yarn dry yarn is wrinkled by introduced buckles that are aligned approximately parallel to the local fiber orientation direction within the coiled yarn, as shown in Fig. 1c,d, and most clearly in Figure S7. This structure provides sufficient space for large yarn volume change upon water absorption without causing damage in the hybrid yarn. As the HYAM absorbs water, the pressure exerted by the PDDA on the twisted CNT host increases, and the volume is reversibly recovered in part through an unfolding of the structure, as shown in Fig. 1e,f. To determine the distribution of PDDA in the HYAM, a cross-section of the HYAM was imaged (Fig. 1g) and elemental mapping analysis was performed, as shown in Fig. 1h and Figure S2. This mapping shows a homogenous distribution in the HYAM of chlorine (which occurs only in the PDDA molecule) on the investigated dimensional scale^22^.

### Self-powered PDDA/CNT hybrid artificial muscle driven by change of moisture content

**Figure 2:**
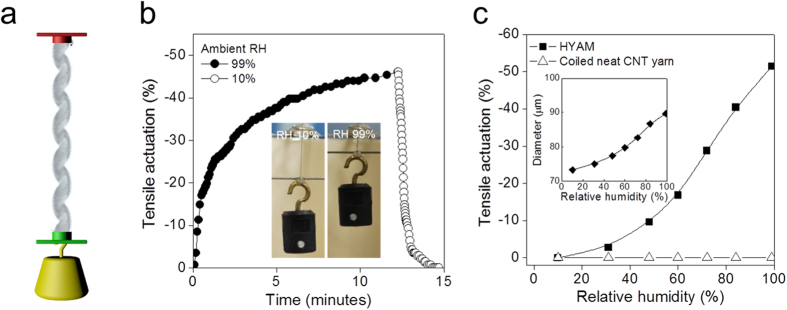
Configuration of the HYAM and tensile actuation driven by RH. The HYAM including 30 wt% PDDA of 67 μm diameter and 20 mm length was characterized under isobaric 2.3 MPa load. (**a**) The configuration of a two-end-tethered HYAM. Red and green yarn-end attachments are tethers, meaning they prohibit end rotation; the red attachment also prohibits translational displacement. (**b**) The time dependence of tensile actuation driven by water absorption and desorption. The tensile contraction and expansion of the HYAM occured in 99% RH (filled circles) and in 10% RH (open circles), respectively. The inset shows photographs of the HYAM before and after contraction to lift the pictured weight. (**c**) The equilibrium tensile actuation of the HYAM (filled squares) and coiled neat CNT yarn (open triangles) versus RH. The inset shows the dependence of the diameter of the HYAM (filled diamonds) on RH.

The configuration for evaluating the mechanical actuation of the HYAM with an applied isobaric load is illustrated in Fig. 2a. The end tethers permitted length changes but prevented net rotation, so that the total number of turns in the coiled yarn was preserved during actuation. However, changes in hybrid yarn twist could be accommodated by decrease of coil twist, so that yarn untwist was converted into a contraction in coil length. Twist or untwist of the yarn within the HYAM occurs as it is exposed to a change in RH, because the RH around the HYAM determines the moisture content in the PDDA and the volume of the hybrid yarn.

When the ambient RH around the HYAM was changed from 10 to 99%, the HYAM slowly delivered a 52% stroke during 12 minutes at an isobaric load of 2.3 MPa. When the RH was changed from 99 to 10%, the HYAM more quickly fully recovered its initial length by water evaporation in 3 minutes (Fig. 2b). Although the time required to absorb water from the air was long, the HYAM eventually delivered an equilibrium tensile contraction of up to 52% for RH change from 10% to 99% (Fig. 2c). This figure additionally shows the dependence of equilibrium tensile stroke on RH. In all tests the initial isobaric load was applied to the hybrid yarn in the dry state, and any creep allowed to disappear before the actuation test commenced. During this water-absorption process the wrinkled structure unfolds and is accompanied by a change in the diameter of yarn from 73.2 to 89.6 μm depending on the RH (inset of Fig. 2c). For comparison, when the pristine coiled CNT yarn was tested under the same conditions, no tensile actuation was observed upon changing the RH.

**Figure 3:**
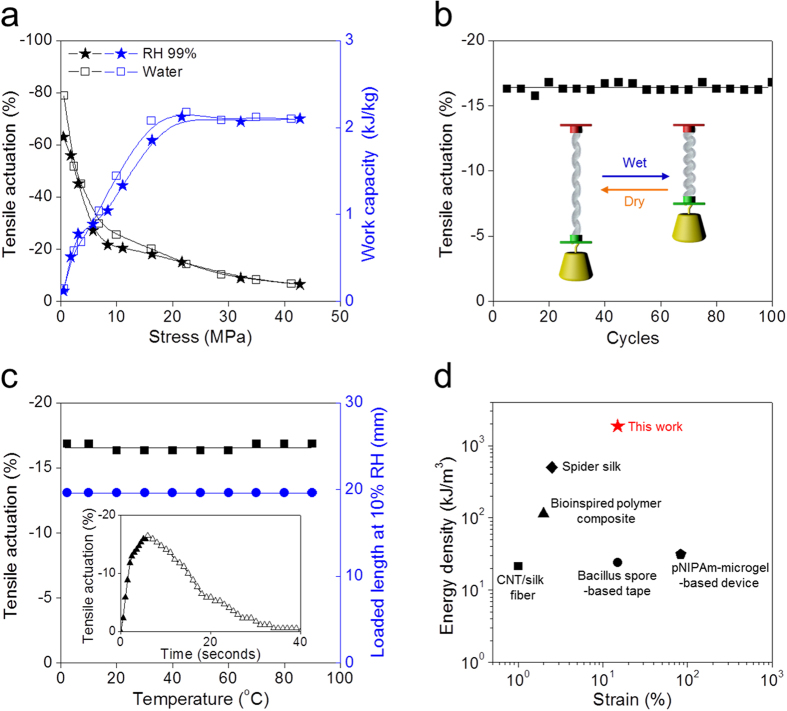
Tensile actuation and work capacity of a HYAM. A 67-μm-diameter (the diameter of the yarn within the coiled structure) HYAM containing 30 wt% PDDA polymer was used. (**a**) The stress dependence of steady-state tensile actuation and contractile work (black and blue symbols, respectively) driven by change in RH (filled stars) from 10 to 99% and contact with liquid water (open squares). (**b**) Tensile actuation stroke (under an isobaric load of 20.7 MPa) for 100 cycles between wet and dry states. The inset schematically illustrates the actuation process. (**c**) Steady-state tensile contraction (black squares) at an isobaric load of 20.7 MPa as a function of water temperature. The dry loaded muscle length (at 10% RH) versus temperature is also shown (blue circles). The inset shows one cycle of muscle contraction at 20 °C (filled triangles during absorbing water) and expansion (open triangles during water evaporation to 10% RH). (**d**) Comparison of the work capacity of tensile moisture driven actuator and stroke for the HYAM muscle of (**a**) (red star) with those for previously reported systems (pNIPAM-microgel based device^8^, *Bacillus* spore based tape^13^, bio-inspired polymer composite^18^, CNT/silk fiber^26^,^27^, and spider silk^28^). Here and elsewhere, tensile stresses are engineering stress values based on the diameter of HYAM fiber in the unloaded state and before water absorption.

There were no significant differences between either the equilibrium tensile stroke or the work capacity obtained by dropping droplet of water on the dry yarn or by RH changes from 10% to 99% (Fig. 3a). As the isobaric load applied to the HYAM was increased from 0.54 to 42 MPa, the tensile stroke decreased from 78 to 6.5%, whereas the work capacity per mass of the HYAM increased from 0.12 to 2.17 kJ kg^−1^. The HYAM held under 22.5 MPa isobaric conditions provided 14.3% tensile stroke (see the Supporting Information, Movie 1) and the maximum 2.17 kJ kg^−1^ work capacity, which is 54 times higher than the work capacity of natural muscle (0.039 kJ kg^−1^). HYAMs fabricated at an applied isobaric load of 5.7 MPa provided a higher work capacity and a larger tensile contraction than HYAMs formed a lower isobaric load of 1.7 MPa (Figure S3). Compared with other reported hybrid muscles based on coiled structures, the work capacity of HYAM is two times higher than that of thermally-actuated CNT yarn filled with paraffin wax (1.36 kJ kg^−1^)^3^ and two times higher than that for a silicone-CNT yarn (1.1 kJ kg^−1^) driven by solvent absorption^24^.

The changes in morphology observed for the HYAM were found to be highly reversible. Importantly, tensile stroke was constant for the investigated 100 cycles between dry and fully wet states under the isobaric mechanical load (20.7 MPa) that provides maximized work capacity (Fig. 3b). Additionally, as shown in Fig. 3c, tensile contraction between 0 to 90 °C was essentially independent of temperature and no creep was observed up to this highest temperature. Actuation of the HYAM was also independent of water pH between pH 3 and pH 11 (Figure S4) and the length of the dry muscle was not significantly affected by temperature changes between 24 °C and 84 °C (Figure S5). A contraction of 14% was induced in 6 seconds by exposing the muscle in liquid water for 6 seconds, and this actuation was reversed in 35 seconds by allowing water evaporation into a 10% RH atmosphere (inset of Fig. 3c). During contraction, the HYAM provided 0.36 kW kg^−1^ of average gravimetric mechanical power density under an isobaric 20.7 MPa load, which is much higher than for either a moisture-driven bending actuator (0.0025 kW kg^−1^)^18^ or a moisture-driven torsional actuator (0.072 kW kg^−1^)^20^. The HYAM also showed fast 3.95% s^−1^ average contraction strain rate under 20.7 MPa while absorbing water. The response rate of the HYAM fibers is nearly the same as the fastest previously reported hygromorph tensile muscles (Table S1), and will decrease with decreasing fiber diameter (Figure S6). Since the carbon nanotube hybrid yarn has a well-aligned structure with a large number of interconnected nanoscale and microscale channels (Figure S7)^15^, these channels enable the fast absorption and desorption of water, as has been demonstrated in previously reported hygromorph muscles^25^. Comparison of the volumetric energy density and actuator stoke of the HYAM with those of reported moisture driven tensile actuators, presented in Fig. 3d, demonstrates that the present HYAM provides the combination of record energy density (1.8 MJ m^−3^) with very high strokes. The HYAM provides 1.8 times higher volumetric energy density than a much denser NiTi shape memory alloy actuator (1 MJ m^−3^), which is driven by thermal energy^26^.

### Demonstration of automatic ventilation system

**Figure 4:**
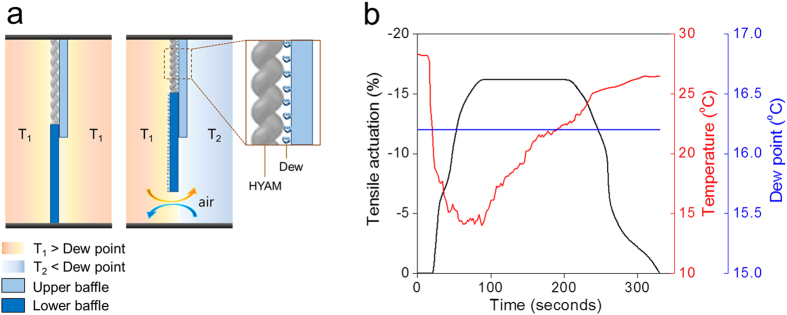
Application in automatic ventilation. Three HYAMs (each 67 μm in diameter and having a 28.3 mm non-loaded length at 50% RH) were used to raise a lower baffle in response to water condensation. The air had 50% RH at 28.4 °C (T_1_) until cooled on one side of the baffles (**a**) Schematic diagram of the automatically controlled device based on condensation-driven contraction of the HYAMs. (**b**) Time trace of tensile actuation depending on the temperature of actuator side of the baffle (measured using a thermocouple), relative to the dew point. The temperature of actuator side of the baffle was controlled by cooling outside of baffle for producing dew. The dew point (16.2 °C) was calculated from the 50% RH at 28.4 °C.

A ventilation system that provides controllable humidity was developed in this study as a demonstration of the possible eventual utility of the hygromorph actuators. Ventilation in daily life is typically controlled by opening and closing vents, windows, or doors, but is not enabled by a self-powered material that intelligently both senses and actuates. In this context, we have designed and demonstrated a crude prototype of a HYAM-based, autonomous, moisture-driven ventilation system that does not require an external energy supply. The working principle and scheme of the ventilation system are schematically shown in Fig. 4a. The upper baffle is fixed on the top plate, and the lower baffle is connected to the end of HYAMs. When either dew appears or the RH increases on the surface of the HYAM, the lower baffle moves up because of automatic water-drive HYAM contraction (Fig. 4a).

We used three 67-μm-diameter, 28-mm-long HYAMs, which were operated at the mechanical load that optimized work capacity (Fig. 3a), to demonstrate this miniature automatic ventilation system. The dependence of the tensile actuation of the HYAMs on the temperature of the HYAM side of the baffle is shown in Fig. 4b. As this temperature of the top baffle decreased to below the dew point (16.2 °C) because of cooling from the cold-side temperature T_2_, dew started to condense on the surface of the baffle. This moisture drove the muscle contraction that lifted the lower baffle. In doing this, the parallel HYAMs contracted in length by 16.2%, thereby lifting a 12 g mass (which is 12,000 times heavier than the total weight of the three HYAMs). When the temperature gradually increased from 14.1 °C to 26.4 °C, the HYAMs expanded as the dew evaporated.

## Discussion

By incorporating poly(diallyldimethylammonium chloride) guest in a coiled carbon nanotube yarn, a large stroke, highly reversible, giant-work-capacity artificial muscle yarn has been demonstrated that is powered by fluctuations in relative humidity or by direct contact with liquid water. The tensile work capacity during muscle contraction and stroke are insensitive to both the pH of the water and temperature, but depend upon the applied mechanical load. For an applied isobaric load of 22.5 MPa, a stroke of 14.3% and a work capacity during contraction of 2.17 kJ kg^−1^ have been demonstrated, which is 5.5 times higher than the work capacity of the previous record holder as a water-powered muscle: Nature’s spider silk. The muscle contraction reaches 78% for an isobaric load of 0.54 MPa, which is twice that of natural skeletal muscle, whose load-lifting capability does not exceed about 0.4 MPa. When increasing the diameter from 50 μm to 150 μm under isobaric 21 MPa load (Figure S6), the hybrid yarns showed roughly constant tensile contraction (∼15%) and work capacity (∼1.9 kJ kg^−1^), while the stoke rate decreased from 3.95% s^−1^ to 0.46% s^−1^. These results illustrate that increasing muscle diameter can lead to undesirable increases in response times. However, unlike the case for previous bending and torsional actuators, scale-up is possible without a decrease in response rate by operating essentially unlimited numbers of well-separated small-diameter tensile HYAMs in parallel.

## Methods

### Materials

Spinnable MWNT forests, composed of approximately 12 nm diameter MWNTs that contain approximately nine walls and form large bundles, were grown on a Si wafer by chemical vapor deposition^23^, and used for drawing the MWNT sheets. Poly(diallyldimethylammonium chloride) solution (20 wt.% in H_2_O) was purchased from Sigma-Aldrich (USA).

### Sample preparation

Coiled PDDA/CNT hybrid yarn muscles were prepared by biscrolling, which involved inserting twist into a carbon nanotube sheet stack that had been infiltrated with PDDA^23^. To infiltrate the PDDA into the CNT sheet stack, the CNT sheet stack (composed of five stacked sheet layers, which were 25 mm wide and 10 cm long) was immersed in the above PDDA solution for 1 minute. After withdrawal from the PDDA solution, the CNT sheet stack included 30 wt% PDDA. One end of the sheet stack was attached to the shaft of a stepper motor and the opposite end was attached to a torsionally tethered weight (which provides an isobaric load of 5.7 MPa). After fully swelling the PDDA in the infiltrated sheet stack with water, the coiled PDDA/CNT hybrid yarn was prepared by inserting 3,700 turns/meter of inserted twist into the CNT sheets (where the turns/meter is normalized by the final non-loaded length of the coiled yarn). After biscrolling, the coiled PDDA/CNT hybrid yarn was dried in room temperature air having 10% RH.

### Characterization

A cross-sectional section of the yarn was prepared using a Focused Ion Beam (FEI Nova 200 NanoLab DualBeam FIB/SEM). This cross-section of the coiled PDDA/CNT HYAM was imaged using a scanning electron microscope (Zeiss SUPRA 40 FE-SEM) and the elemental composition was analyzed using an EDAX Lithium detector. The morphology of the PDDA/CNT HYAM was observed by FE-SEM using a Low Vacuum & Bio Application Technology instrument (Quanta 250 FEG).

### Calculation of mechanical properties

The tensile stroke of the HYAM was calculated^3^,^4^ using equation (1)


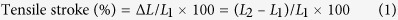


where *L*_*1*_ is the initial length of HYAM at 10% RH under isobaric load and *L*_*2*_ is the final length at ambient RH under isobaric load. The work capacity per mass of the HYAM was calculated by dividing the work of contraction (kJ) by the mass (kg) of the actuating coiled fiber^3^,^4^. The equation used for this calculation is


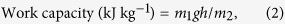


where *m*_*1*_ is isobaric load (kg), *g* is gravitational acceleration, *h* is the change of height with isobaric load, and *m*_*2*_ is the mass of the HYAM, which does not including the mass of water.

The volumetric energy density is defined by dividing the work during contraction (kJ) by the volume (m^3^) of actuating fiber^17^. The equation used for this calculation is energy density is:


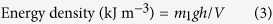


where *V* is volume (m^3^) of the HYAM. The HYAM volume was obtained by multiplying cross-sectional area (using the outer coil diameter of the HYAM) and its length at 10% RH.

## Additional Information

**How to cite this article**: Kim, S. H. *et al.* Bio-inspired, Moisture-Powered Hybrid Carbon Nanotube Yarn Muscles. *Sci. Rep.* **6**, 23016; doi: 10.1038/srep23016 (2016).

## Supplementary Material

Supplementary Information

Supplementary Video
