# Supplementary material for: Bio-inspired, Moisture-Powered Hybrid Carbon Nanotube Yarn Muscles
Source: Sci Rep. 2016 Mar 14;6:23016. doi: 10.1038/srep23016 (PMC4789747; doi:10.1038/srep23016)
Supplement: Supplementary Information [file srep23016-s1.pdf]

Supporting Information

Bio-inspired Artificial Muscle of Hybrid Carbon Nanotube Yarn Driven by Moisture

Shi Hyeong Kim,[1] Cheong Hoon Kwon,[1] Karam Park,[1] Tae Jin Mun,[1] Xavier Lepro,[2] Ray H. Baughman,[2] Geoffrey M. Spinks,[3] and Seon Jeong Kim[,1]*

[1]Center for Self-powered Actuation and Department of Biomedical Engineering, Hanyang University, Seoul 133-791, South Korea.

[2]The Alan G. MacDiarmid NanoTech Institute, University of Texas at Dallas, Richardson, TX 75083, USA

[3]Intelligent Polymer Research Institute, ARC Centre of Excellence for Electromaterials Science, University of Wollongong, Wollongong, New South Wales 2522, Australia

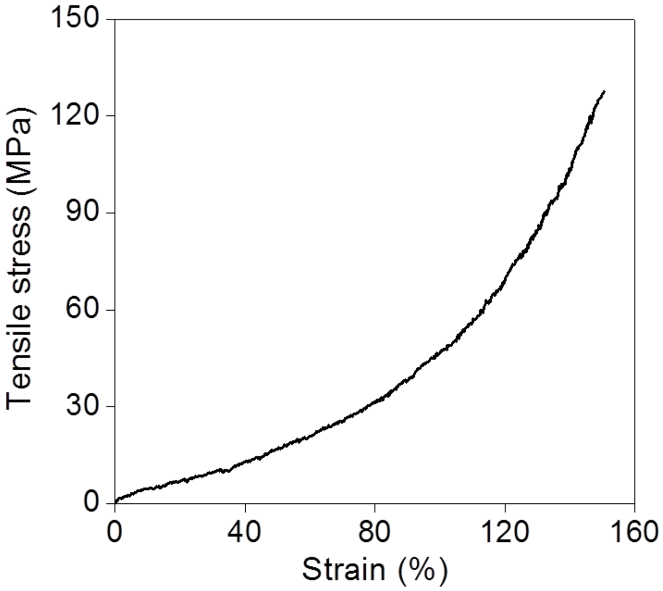

Figure S1. Stress-strain curve obtained for a coiled hybrid yarn that was initially 17 mm long and 70 μm in diameter. This dry fiber had a breaking strain of 150% and an elastic modulus of 32 MPa in the low strain region.

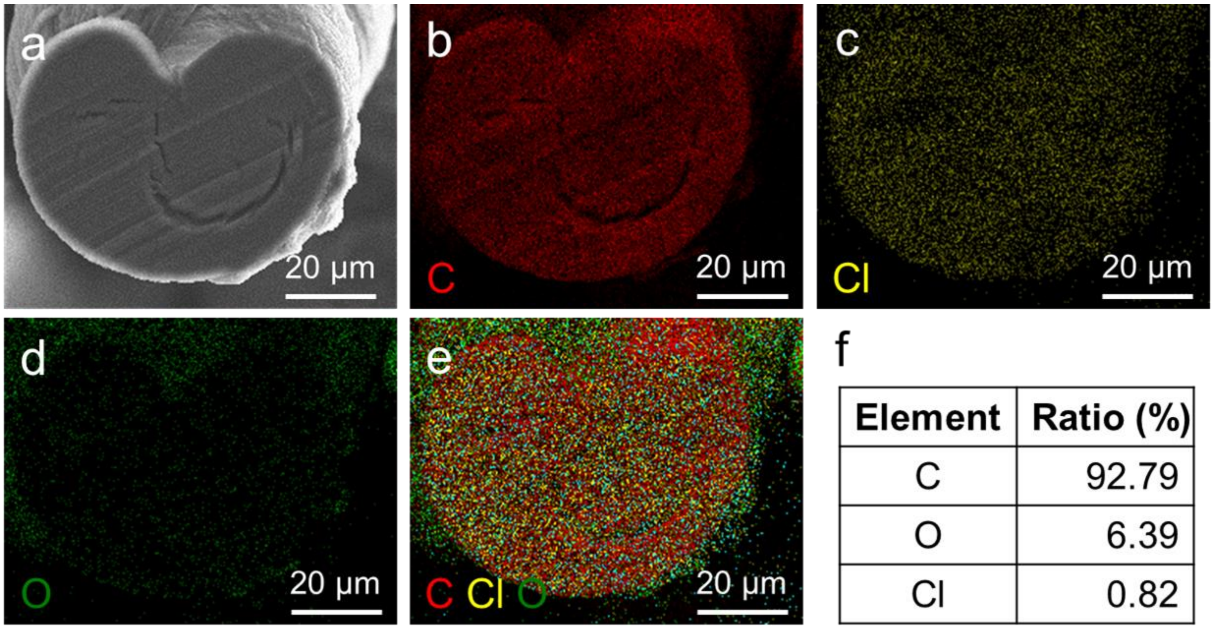

Element	Ratio (%)
C	92.79
O	6.39
Cl	0.82

Figure S2. Element mapping by EDAX. (a) Cross-sectional SEM image of the HYAM. (b)-(d) Images showing carbon, chloride, and oxygen concentrations, respectively. (e) Overlap image of the concentrations shown in the images of (b)-(d). (f) Table showing the average ratio of elements that is derived from the EDAX.

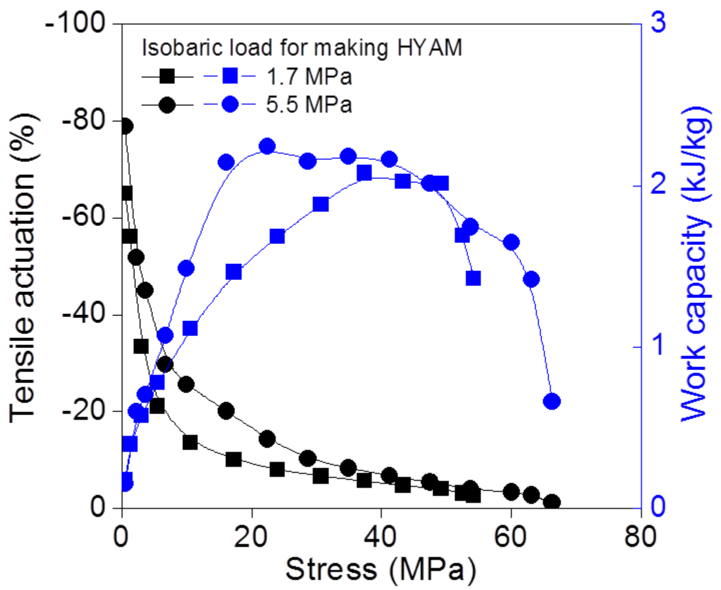

Figure S3. Influence of the isobaric load applied during twist spinning the coiled yarn on the dependence of tensile actuation and work capacity on applied tensile stress. The HYAM made using an isobaric load of 5.5 MPa during twist insertion provided the highest work capacity during contraction (2.17 kJ/kg).

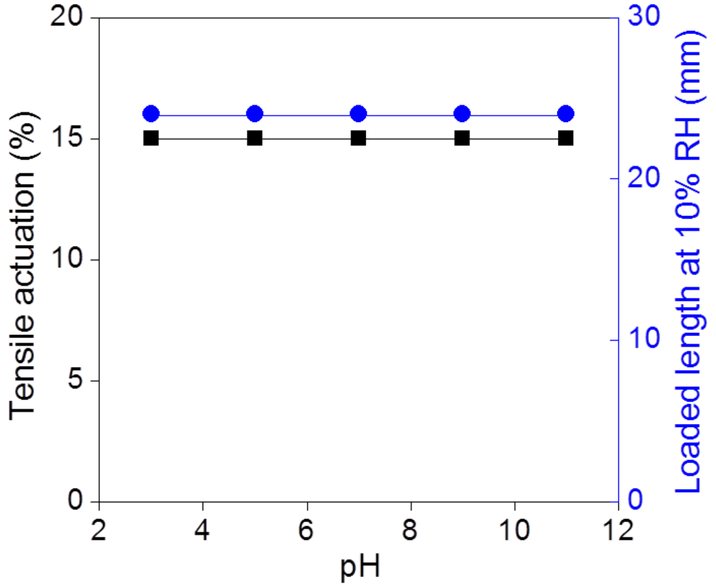

Figure S4. The dependence of tensile actuation and loaded length of a hybrid yarn on water pH, when the applied load was 21 MPa. The muscle, when under this load, was 24 mm long and 80 µm in diameter for a 10% RH. The water pH was controlled by addition of a HCl solution for acidic conditions and a NaOH solution for alkali conditions. As shown here, the tensile actuation (black square) and length (blue circle) of the hybrid yarn was not affected by pH.

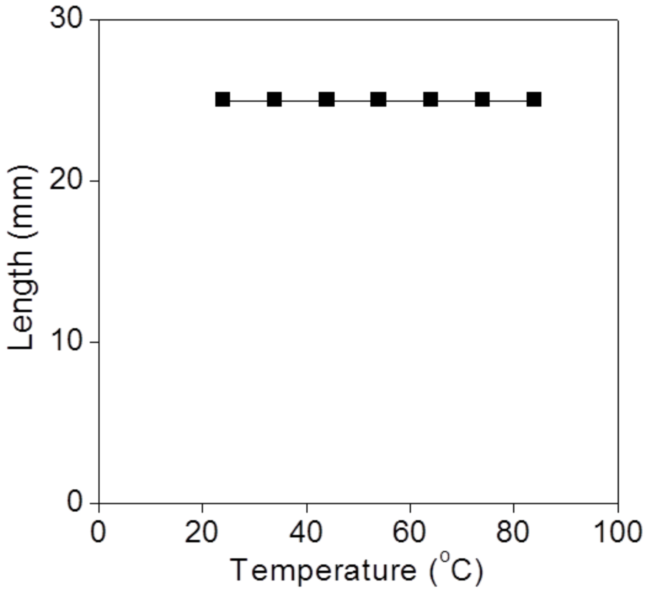

Figure S5. The dependence of the length of a dry hybrid yarn on temperature when the applied load was 21 MPa. At 20 °C and 10% RH. the muscle was 24 mm long and 80 μm in diameter.

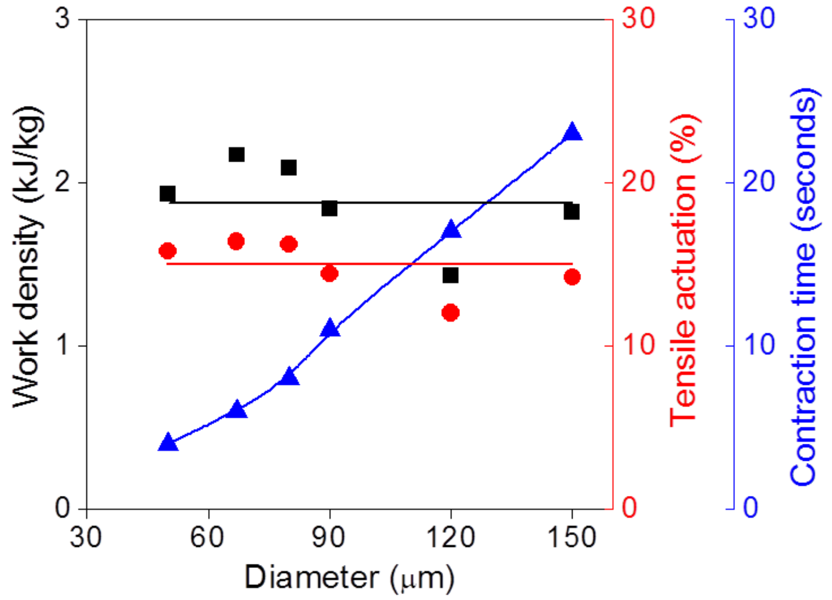

S6. The work density (black square) during contraction, tensile actuator stroke (red circle)and contraction time (blue triangle) as a function of yarn diameter. Actuation was between dry (10 % humidity) and fully wet states.

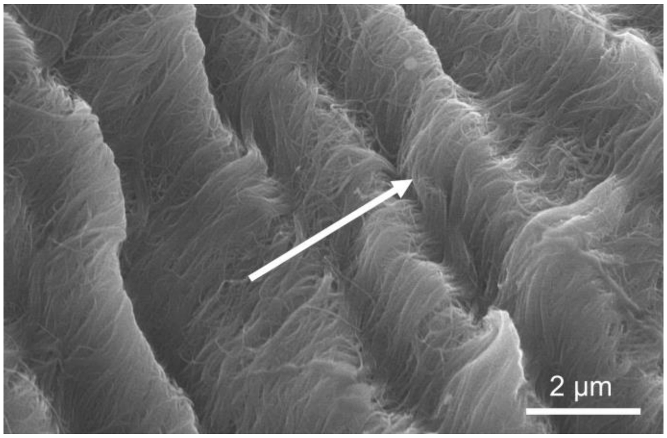

Figure S7. SEM image of the surface of a dry PDDA/CNT hybrid yarn, where the long and short white arrows indicate the direction of the coiled yarn's length and the local direction of the yarn within a coil.

Table S1. Comparison of tensile strain and response rates of various hygromorph tensile muscles.

Materials	Stimulus	Diameter (μm)	Tensile strain (%)	Response time (seconds)	Response rate ($\% \, s^{-1}$)	Ref.
PDDACNT hybrid yarn (Our work)	Water	50	15.8	4	3.95	
	RH: 10% - 99%	67	52	720	0.72	
Graphene oxide fiber	RH: 20% - 80%	34	5	10	0.5	9
Graphene hydrogel fiber	RH: 20% - 85%	62	4.7	12	0.4	20
CNT/silk fiber	RH: 0% - 70%	6.5	0.4	240	0.0017	27
Spider silk	RH: 10% - 90%	5	0.5	2	0.025	28
